# Evaluation of serum biomarkers after intra-articular injection of rat bone marrow-derived mesenchymal stem cells in a rat model of knee osteoarthritis

**DOI:** 10.1016/j.heliyon.2024.e39940

**Published:** 2024-10-29

**Authors:** Abdulwahab Noorwali, Fadwa Aljoud, Amani Alghamdi, Noora Sattami, Taghreed Bashah, Abdulsalam Noorwali, Peter Natesan Pushparaj, Kalamegam Gauthaman

**Affiliations:** a Regenerative Medicine Unit, King Fahd Medical Research Centre, King Abdulaziz University, Jeddah, 21589, Saudi Arabia; b Institute of Genomic Medicine Sciences (IGMS) and Department of Medical Laboratory Technology, Faculty of Applied Medical Sciences, King Abdulaziz University, Jeddah, 21589, Saudi Arabia; c Center for Transdisciplinary Research, Department of Pharmacology, Saveetha Dental College and Hospital, Saveetha Institute of Medical and Technical Sciences, Saveetha University, Chennai, 600077, India; d Pharmaceutical Division, Nibblen Life Sciences Private Limited, Chennai, 600061, India; e Scientific Research Center, Dar Al-Hekma University, Jeddah, 22246, Saudi Arabia

**Keywords:** Osteoarthritis, Biomarkers, Mesenchymal stem cells, Histomorphometry, Ingenuity pathway analysis

## Abstract

**Background:**

Osteoarthritis (OA) is a prevalent joint disorder characterized by joint pain, functional impairment, and disability. The current study investigated the therapeutic effects of intra-articular injection of rat bone marrow-derived mesenchymal stem cells (rBM-MSCs) in rats with knee OA.

**Methods:**

Fourty five male Wistar rats were randomly divided into three groups (A-C) and received either an intra-articular injection of normal saline (NS) or rBM-MSCs. The normal control group (A, n = 15) received NS, the OA control group (B, n = 15) received NS, and the OA treated group (C, n = 15) received rBM-MSCs (0.5 × 10^6^ cells in 25 μL NS). Knee OA was induced using monosodium iodoacetate (MIA). rBM-MSCs were sourced from female Wistar rats and their stem cells were characterized using flow cytometry. Histomorphometric analyses were performed on knee sections from both normal and OA knee. Serum biomarkers, including hyaluronic acid (HA), cross-linked N-telopeptide of type I collagen-1 (NTX-1), NGF, calcitonin gene-related peptide (CGRP), matrix metalloproteinase-3 (MMP-3), oligomeric cartilage matrix protein COMP, interleukin-6 (IL-6), and soluble IL-6 receptor (sIL-6R), were analyzed using ELISA kits. Ingenuity Pathway Analysis (IPA) was used to determine the genes regulated by MSCs in OA, and the protective mechanisms were determined using the Molecular Activity Predictor (MAP).

**Results:**

rBM-MSCs were positive for CD29 and CD90 and negative for CD45 surface markers. OA biomarkers were significantly elevated in the untreated OA group but decreased after treatment with intra-articular MSCs. The OA group treated with MSCs showed significant repair of the damaged cartilage compared to the control group.

**Conclusions:**

Cartilage damage leads to an increase in inflammatory cytokine levels and is associated with an increase in serum biomarkers related to cartilage degradation. Intra-articular administration of MSCs showed beneficial effects, including regeneration of damaged cartilage and a reduction in inflammation-related serum biomarker levels.

## Graphical abstract

**Figure:**
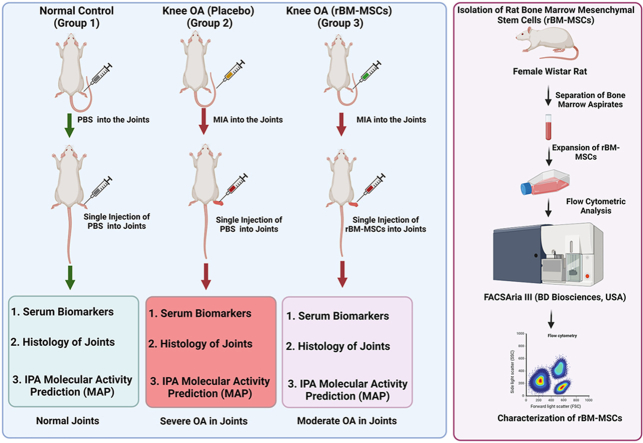

## 1 Introduction

Osteoarthritis (OA) is a degenerative joint disease and a leading cause of chronic disabilities worldwide, particularly affecting the elderly population [[1], [2], [3]]. The knee, hip, hand, and back joints are the most commonly affected in OA [4,5]. The prevalence of OA is estimated to be 80 % in individuals over 65 years of age [6] and is considered a leading cause of physical disability, ranking 11th in the World Health Organization's (WHO) Global Burden of Disease [[7], [8], [9]]. OA is a significant contributor to the economic burden on public health systems, particularly in the United States, where the annual cost of knee and hip replacement surgery was estimated to be $42 billion in 2009 [10]. The indirect costs of OA are primarily attributed to reduced productivity and absenteeism from work, with a UK study estimating the cost of reduced or absent productivity of OA patients at £3.2 billion and the cost of community and social services at £258 million [11,12]. Current treatments for OA aim to alleviate symptoms, control pain, and improve patients' quality of life, but unfortunately, these interventions cannot prevent joint wear or halt the progression of OA [13,14].

Mesenchymal stem cells (MSCs) are multipotent stem cells that can be found in various tissues throughout the body, such as bone marrow, adipose tissue, placenta, umbilical cord, and synovium [15]. These cells are capable of differentiating into various cell types including chondrocytes, adipocytes, and osteoblasts [16]. MSCs possess immunosuppressive properties, which have been shown to help alleviate inflammation associated with OA [17]. As a result, MSCs could be useful in the treatment of OA as they can promote the healing of damaged cartilage and support its regeneration [18]. Intra-articular injection of MSCs has been reported to improve pain and function in several preclinical and clinical studies [13,18]. Additionally, recent studies have demonstrated that cartilage volume can regrow and change after MSC injections.

Biomarkers play a crucial role in the early detection and follow-up of treatment for OA. Their identification allows for the assessment of tissue repair and regeneration. Validated biomarkers include oligomeric cartilage matrix protein (COMP), matrix metalloproteinases (MMPs), calcitonin gene-related peptide (CGRP), hyaluronic acid (HA), cross-linked N-telopeptide of type I collagen (NTX-1), and nerve growth factor (NGF) [[19], [20], [21], [22], [23], [24], [25]]. The proinflammatory cytokine interleukin-6 (IL-6) is elevated in the serum of OA patients and promotes the chondrogenic differentiation of MSCs through the soluble IL-6 receptor (sIL-6R) [25].

In this study, we investigated the therapeutic effects of intra-articular injection of rBM-MSCs in rats with knee OA and evaluated the efficacy of rBM-MSC therapy using various biomarkers associated with the progression of cartilage degeneration, inflammation, and pain. Our study aimed to provide insights into the potential benefits of rBM-MSCs as a therapeutic intervention for OA.

## 2 Materials and methods

### 2.1 Animals

In vivo experiments were conducted on male Wistar albino rats (n = 45), aged eight to ten weeks and weighing an average of 250–300 g. Female Wistar albino rats (n = 10), aged four to six weeks and weighing an average of 120–150 g, were utilized to obtain MSCs from the bone marrow. This study was approved by the Bioethics Committee of King Abdulaziz University (KAU), Jeddah, Saudi Arabia, under protocol number (374-16). All animal experiments were carried out in accordance with the guidelines of our institution and the Animal Welfare Committee. Knee OA was induced in 30 rats by administering monosodium iodoacetate (MIA) as described previously [[26], [27], [28]]. Each animal received an intra-articular injection of MIA (2 mg/25 μl) into the femorotibial joint space of the left hindlimb, 14 days prior to the experiment (Fig. S1).

### 2.2 Preparation of mesenchymal stem cells

Mesenchymal stem cells (MSCs) were obtained from 10 female Wistar rats weighing 120–150 g. The rats were euthanized using inhalational overdose of carbon dioxide. The long bones of rats (tibia and femorae) were collected and immediately transferred to the laboratory under sterile conditions. The bone cavities were repeatedly flushed using sterile saline to extract the bone marrow contents. The collected marrow contents were passed through successive smaller-size needles, and centrifued at 100 rpm for 10 min. The pellet was reconstitued in culture medium and plated in T 25 cm^2^ tissue culture flasks. The culture medium comprised of high-glucose Dulbecco's modified Eagle's medium (DMEM-HG) containing 2 mM glutamine and 1 % antibiotic/antibody solution. The flask and its contents were maintained under standard conditions at 37 °C in an incubator with 5 % carbon dioxide and 95 % atmospheric air. The dead and floating cells were later removed with media changes and the adherent cells were expanded and analyzed using flow cytometry (FACS Aria III, BD Biosciences, USA) to determine the expression of cell surface markers associated with MSCs, specifically CD29 and CD90, and to exclude the presence of CD45, a marker for hematopoietic cells as perviously described [29].

The experimental design involved the utilization of 45 rats, which were randomly assigned into three distinct groups, with 15 rats in each group. Group-A served as the normal control and received a single intra-articular injection of normal saline (NS; 25 μl). Group-B constituted the OA control, and also received a single intra-articular injection of NS (25 μl). Lastly, Group-C was designated as the OA treatment group, and received a single intra-articular injection of rBM-MSCs (0.5 × 10^6^ cells in 25 μl NS). Five rats from each group were sacrificed at 10, 20, and 30 days of observation, and blood samples and knee joints were collected for biochemical and histomorphometric analyses respectively.

### 2.3 Serum collection

Blood samples (5 ml) were collected from all study groups using the retro-orbital sinus method using light anesthesia with diethyl ether. Following collection, the samples were allowed to clot at room temperature for 30 min, and then centrifuged at 3500 rpm for 15 min. The separated serum was collected and stored in aliquots at −80 °C until further analysis.

### 2.4 Biomarker analysis

Serum levels of the following biomarkers were evaluated using the kits purchased from Elabscience (Houston, Texas, USA), and serum biomarker levels were determined by enzyme-linked immunosorbent assay (ELISA) according to the manufacturer's instructions for each kit.: HA (catalog number: E-EL-0036), NTX-1 (catalog number: E-EL-R0276), NGF (catalog number: E-EL-R0652, 96T), CGRP (catalog number: E-EL-R0135, 96T), MMP-3 (catalog number: E-EL-R0619, 96T), COMP (catalog number: E-EL-R0159, 96T), IL-6 (catalog number: E-EL-R0015), and Sil-6R (catalog number: E-EL-R0896).

### 2.5 Histopathology

For the histological and morphometric analyses of the knee joints, both control and OA rats were euthanized on the 10th, 20th, and 30th experimental days, and the joints were dissected from the soft tissues on both sides of the joints. The joints were then fixed in 10 % formalin for 24 h, decalcified in a tissue decalcifier (TBD-1, Thermo Scientific, USA), and sectioned in both the frontal and sagittal planes. The sections were then embedded in paraffin blocks and tissue sections (10 μm) were prepared and mounted on glass slides. The tissue sections were stained with hematoxylin and eosin and examined under a light microscope for cartilage pattern and/or regeneration in experimental and control rats. To assess osteoarthritic changes in the stained sections, the following criteria were examined and compared qualitatively between the control and treatment groups: surface irregularities, organization and hypertrophy of chondrocytes, and degree of proliferation and inflammation of synovial cells.

### 2.6 Ingenuity Pathway Analysis of genes regulated by mesenchymal stem cells in osteoarthritis

The genes involved in the functional regulation of the biological, cellular, and molecular functions of MSCs that are relevant to OA were obtained from the Ingenuity Pathway Analysis (IPA) Knowledgebase (Qiagen, USA). IPA core analysis was implemented to identify the various canonical pathways, upstream regulators, causal networks, diseases and biofunctions, undirected networks, and pathological functions regulated by MSCs based on Fisher's exact test (P < 0.05) [[30], [31], [32]]. Key genes involved in the regulation of OA, connective tissue development, and disease were identified using IPA. Furthermore, the Molecular Activity Predictor (MAP) tool was used to decipher the protective mechanism of MSCs in OA [33].

### 2.7 Statistical analysis

The levels of serum biomarkers in the control and treatment groups were examined through one-way Analysis of Variance (ANOVA). To compare the serum biomarkers between the Control OA group at day 0 and the rBM-MSCs group at days 10, 20, and 30, a student's t-test (two tailed) was performed. The data is presented in terms of the mean ± standard deviation (SD) of three separate experimental replicates. A P value of less than 0.05 was considered statistically significant.

## 3 Results

### 3.1 Characterization of MSCs in culture: Isolation, expansion, and surface marker analysis

**Fig. 1:**
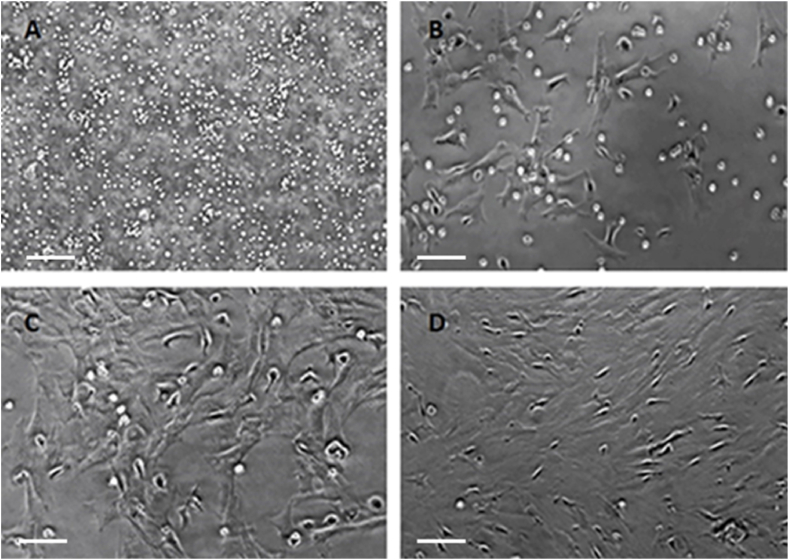
Phase contrast image of rat bone marrow mesenchymal stem cells (rBM-MSCs). A) Day 0 – Plated bone marrow aspirate with floating red blood cells; B) Day 3 – monolayer of adherent cells resembling small fibroblasts and stellate cells; C) Day 7 – increase in cell number with flattened and elongated fibroblast shape; D) – confluent rBM-MSCs at day 7 following subpassage (P2). Scale Bar = 100 μm. Magnification 20×.

When rat bone marrow aspirates were plated in tissue culture flasks, cellular attachment and colony-like expansion were observed within five to seven days. The cells exhibited characteristics of fibroblasts and demonstrated excellent expansion potential after subculturing (Fig. 1A–D).

**Fig. 2:**
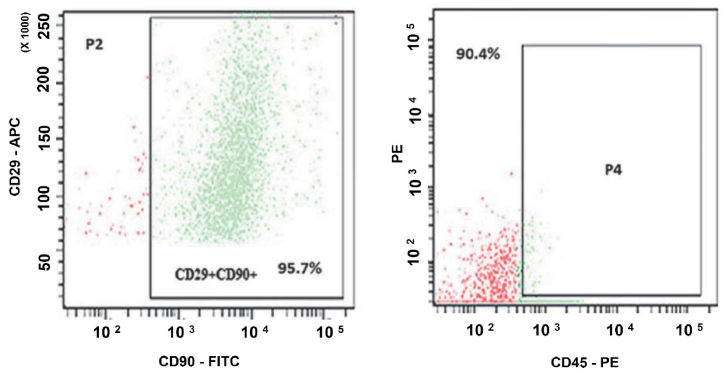
CD Marker characterization of rat bone marrow mesenchymal stem cells (rBM-MSCs). The stemness of the isolated rBM-MSCs was evaluated by fluorescence-activated cell sorting after labeling the cells for various CD markers with different fluorochromes. The isolated rBM-MSCs were positive for CD 29 and CD 90, the positive CD markers related to MSCs. Cells were predominantly negative for the MSC-negative CD marker 45. Abbreviations: CD - cluster of differentiation; APC - allophycocyanin; FITC - fluorescein isothiocyanate; PE - phycoerythrin.

Cells cultured in vitro expressed the cell surface markers CD29 and CD90, which are characteristic of MSCs. These cells exhibited minimal expression of CD45 (Fig. 2), indicating their potential to be MSCs.

### 3.2 Expression of serum biomarkers in normal and rBM-MSC-treated OA rats

All serum biomarkers examined showed significantly increased levels in saline-treated OA rats (Group-B) and rBM-MSC-treated rats (Group-C) compared with normal controls (Group-A). However, these biomarkers showed a significant decrease in Group-C compared with Group-B (Table 1).

**Table 1:** Serum biomarkers in normal and osteoarthritis (OA) rats^a^.

Biomarkers	Experimental groups (Albino Wistar Rats; n = 15/group)			P Value
	Group A Normal Control (NS)	Group B OA Control (NS)	Group C (OA and rMSCs)	
**HA**	4.66 ± 1.26	37.37 ± 3.95	15.81 ± 10.08	<0.000∗∗
**NTX-1**	31.56 ± 6.49	90.78 ± 18.82	64.97 ± 9.72	<0.000∗∗
**NGF**	568.54 ± 104.81	1644.13 ± 264.59	1351.68 ± 255.79	<0.000∗∗
**CGRP**	106.26 ± 8.53	256.34 ± 74.73	189.92 ± 62.46	<0.000∗∗
**COMP**	56.57 ± 4.42	74.04 ± 5.10	63.88 ± 3.86	<0.000∗∗
**MMP3**	11.71 ± 0.15	12.93 ± 0.59	11.21 ± 0.41	<0.000∗∗
**IL-6**	121.73 ± 78.12	235.25 ± 56.58	165.24 ± 35.25	<0.000∗∗

a Several serum biomarkers associated with OA were studied in both normal rats and rats with monosodium iodoacetate-induced knee OA. Normal control rats (Group-A) and OA control rats (Group-B) were intra-articularly administered normal saline (NS; 25 μL). Rats in Group-C were treated with intra-articular rMSCs (0.5 × 10^6^ cells in 25 μL NS). The serum samples obtained from each group on different days were pooled before ELISA. Data are presented as mean ± standard deviation (SD) of three different experimental replicates. The P value was considered significant at p < 0.05.

Serum biomarkers at 10, 20, and 30 days in both the treated and control groups are shown in Table 2. The data showed a significant time-dependent decrease in all biomarkers in Group-C compared with the NS-treated OA group. These results indicate the beneficial effect of MSCs in reducing inflammation caused by OA and contributing to the regeneration of damaged cartilage tissue.

**Table 2:** The serum biomarkers at 10, 20, and 30 days in rBM-MSCs treated OA rats compared to control OA rats treated with normal saline at day 0.

Biomarkers	Control OA (n = 5)	10 days (n = 5)	20 days (n = 5)	30 days (n = 5)
HA	35.69 ± 4.45	20.27 ± 6.33∗	16.41 ± 15.08	10.76 ± 5.59∗∗
NTX-1	89.61 ± 10.82	72.33 ± 6.35	65.03 ± 4.68∗	56.81 ± 10.85∗∗
NGF	1548.53 ± 108.93	1149.18 ± 138.42∗	1332.41 ± 173.69	1573.44 ± 257.86
CGRP	242.39 ± 53.01	152.73 ± 24.81∗	195.28 ± 49.91	221.76 ± 86.68
COMP	79.95 ± 1.99	67.54 ± 0.56∗	65.15 ± 0.97∗	58.97 ± 1.41∗∗
MMP3	13.64 ± 0.45	12.46 ± 0.18∗	12.10 ± 0.41∗	12.08 ± 0.53∗
IL-6	267.89 ± 16.60	171.74 ± 28.30∗	149.56 ± 22.74∗	122.22 ± 36.83∗∗

Data are presented as mean ± SD of three different experimental replicates. P value is significant when compared with correspnding control OA group (∗p < 0.05, ∗∗p < 0.01).

### 3.3 Histopathological results

**Fig. 3:**
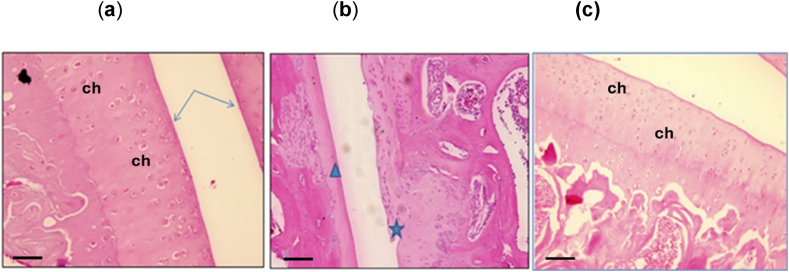
Hematoxylin and eosin (H&E) staining. (a) In the normal control group, the cartilage surface is regular and smooth (arrows), and the chondrocytes (ch) are mostly solitary, uniform in size, and regularly arranged linearly in all layers (b) In the untreated osteoarthritis (OA) group, irregular interface with ingrowth of vascularized connective tissue into the cartilage (arrows). Loss of surface cartilage with fibrosis of the surface (asterisk) and focal marked the thinning of the cartilage (arrowhead) (c) In the OA group treated with rBM-MSCs, diffuse regular thickening of the cartilage with a large number of chondrocytes (ch) in all layers in a linear regular pattern. The interface is regular, and the bone trabeculae appear normal (Scale Bar = 10 μm; Magnification ×100).

H&E-stained sections of cartilage tissue from the normal control rats (group A) displayed the typical features of normal cartilage tissue, including a continuous smooth lining of the articular surface, an intact tidemark, and normal chondrocyte distribution and orientation (Fig. 3A). The cartilage tissue of the OA control rats exhibited varying degrees of cartilage destruction, which became more pronounced at the 20- and 30-day marks and resulted in a significant reduction or obliteration of the joint space, particularly at 30 days.

Intra-articular injection of MIA into the knee joints caused cartilage destruction and loss of articular cartilage integrity, resulting in fragmentation and fissuring of the cartilage matrix. Furthermore, the chondrocytes appeared necrotic and were largely scattered. The matrix deviated from normal staining and appeared acidophilic, especially after 30 days (Fig. 3B).

The MSC-treated OA group demonstrated a near-normal articular surface with regular or smooth cartilage, increased chondrocyte proliferation, and complete recovery of normal histological cartilage characteristics, particularly after 30 days (Fig. 3C).

Histopathological analysis of cartilage tissue from the untreated OA group revealed varying degrees of joint degeneration, including an irregularly thick articular surface with significant loss of articular cartilage integrity, an irregular interface with the bone marrow, and ingrowth of vascularized tissue. Chondrocytes were scattered and necrotic, and no signs of swamping were observed. However, treatment with MSCs resulted in the restoration of the damaged articular surface and the regeneration of articular cartilage, including increased proliferation of chondrocytes with a typical linear pattern and a regular or smooth surface. There was an increase in the proliferation of chondrocytes exhibiting a typical linear pattern, with normal interface with subchondral bone trabeculae, and the regeneration of articular cartilage.

### 3.4 Ingenuity Pathway Analysis of genes involved in mesenchymal stem cells

IPA core analysis of the genes involved in MSCs uncovered diverse canonical signaling pathways, upstream regulators, causal networks, diseases and biofunctions, undirected networks, and pathological functions. Specifically, 28 MSC-related genes were found to play critical roles in osteoarthritis (Table S1). Furthermore, genes associated with connective tissue development and dysfunction were found to be instrumental in biological functions related to chondrogenesis, connective tissue cell development and differentiation, and osteoblast growth and differentiation (Table S2).

**Fig. 4:**
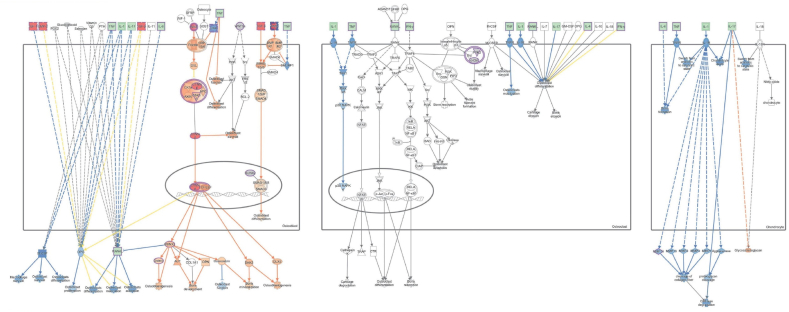
Ingenuity Pathway Analysis of serum biomarkers in the regulation of arthritis using Molecule Activity Prediction tool. The reduction of serum biomarkers such as IL-6, MMP3, etc. attenuates the functions of osteoclasts, osteoblasts, and chondrocytes (marked in blue) and can potentially reduce cartilage damage in OA joints.

**Fig. 5:**
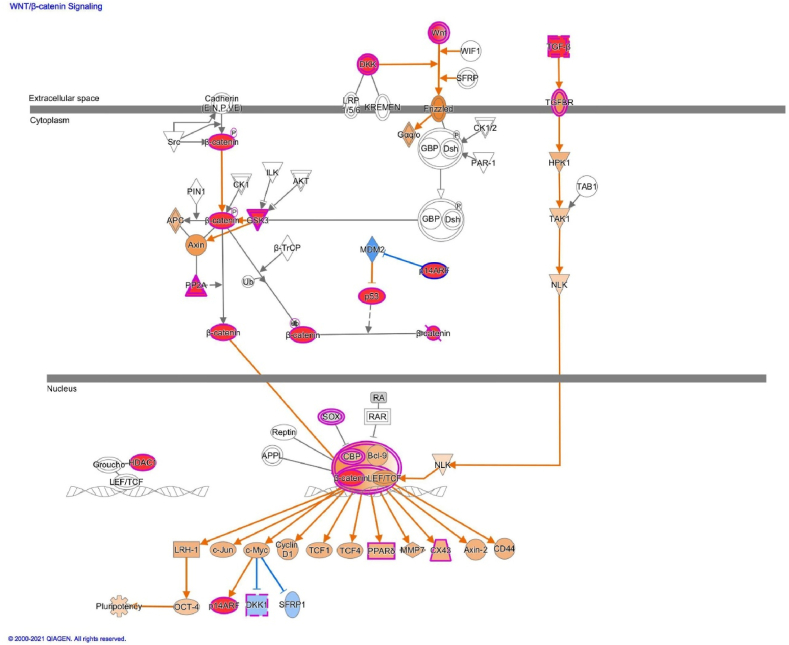
Ingenuity Pathway Analysis of genes implicated in Wnt/beta-Catenin Signaling in mesenchymal stem cells (MSCs) using MAP tool. The analysis of genes implicated in osteoarthritis using the IPA MAP tool showed that Wnt/beta-Catenin signaling was essential for the maintenance of MSC pluripotency.

Downregulation of IL-6 and other inflammatory cytokines using the MAP tool in IPA was found to potentially attenuate the function of osteoclasts, osteoblasts, and chondrocytes in osteoarthritis (Fig. 4). Additionally, transforming growth factor-beta, phosphatase, and tensin homolog deleted on chromosome 10, fibroblast growth factor 2, insulin-like growth factor-1, and β-catenin were identified as the primary upstream regulators (Fig. 5).

## 4 Discussion

Current treatments for OA aim to alleviate symptoms and enhance the quality of life but are largely unable to halt the progression of the disease or reverse underlying joint degeneration. MSCs have the inherent ability to self-renew, making them promising candidates for the treatment of cartilage and subchondral bone defects associated with OA [34]. Several studies have demonstrated the positive effects of intra-articular injection of bone marrow-derived MSCs in various animal models of OA, showing improved cartilage repair and morphological changes [35]. Additionally, animal models of OA treated with MSC have demonstrated significant regeneration of the medial meniscus, reduction in subchondral bone sclerosis and osteophytic remodeling [36,37].

This study investigated the beneficial effects of rBM-MSCs on knee OA in rats, and their correlation with OA-related serum biomarkers, histological assessment of articular cartilage tissue repair, MSC-associated genes, and canonical pathways in OA using bioinformatic tools.

Articular/cartilage degeneration can result in the release of hyaluronic acid (HA) into the bloodstream, a similar phenomenon to osteoarthritis (OA) [38]. N-terminal telopeptides of type-I collagen (NTX-I) is a breakdown product of bone-derived collagen type-I during resorption by osteoclasts and serves as a marker for subchondral bone turnover and/or resorption, as described previously [39]. Both HA and NTX-I are valuable markers for predicting the progression of the disease. In the current study, the increased levels of HA and NTX-I following OA induction in rats and their slight to moderate decrease at various time points (10, 20, and 30 days) after treatment with rBM-MSCs are consistent with previous reports [40,41]. The lack of complete return of HA and NTX-I to normal serum levels after treatment with rBM-MSCs may be due to the persistent effects of MIA. Nonetheless, these serum biomarkers are useful for assessing the severity and therapeutic efficacy of OA.

COMP, a native component of the articular cartilage matrix, can be synthesized by chondrocytes and synovial cells in response to inflammation, as reported previously [19,42]. Matrix metalloproteinase-3 (MMP-3), a member of the MMP family, is involved in the degradation of proteoglycan and laminin, which contribute to the pathogenesis of OA by reducing the elasticity and lubricity of the articular surface. The induction of OA in rats is associated with elevated serum levels of COMP and MMP-3, which were reduced by rBM-MSC treatment. This finding was confirmed by a previous study on OA in rabbits, where the administration of MSCs reversed the serum levels of COMP and MMP-3 [43].

IL-6 is a major pro-inflammatory cytokine within the IL-6 family, which is signaled via glycoprotein 130 (gp130) and membrane-bound/soluble IL-6 receptor (IL-6R) [44]. As previously reported, serum levels of IL-6 and sIL-6R were significantly elevated in the OA group compared to normal levels [24]. Activation of classical (IL-6/IL-6R) or trans signaling (IL-6/sIL6R) occurs via gp130 and contributes to the sequestration of excess IL-6; blocking trans-signaling is effective in preclinical chronic and autoimmune diseases [45]. In addition, the injection of two doses of allogeneic MSCs (0.5 x10^6^ cells) into the joint cavity on days 1 and 3 after the induction of knee OA in dogs resulted in a decrease in IL-6, IL-7, and TNF-α levels, further enhancing the cytokine regulatory effect of MSCs in OA [46].

Elevated levels of NGF have been observed in peripheral tissues of various inflammatory diseases and experimental animal models. Chondrocytes in human osteoarthritis express NGF, and its level positively correlates with the degree of cartilage damage [47]. The untreated OA group in the present study showed high levels of NGF, which is consistent with previous findings that high NGF expression is associated with OA [48]. NGF-positive macrophages and fibroblasts have been observed in the synovial mucosa of patients with knee OA, and their numbers were increased in individuals with advanced OA compared to healthy controls [49]. This may explain the increase in NGF levels in the untreated OA group.

CGRP and NGF are sensory neuropeptides and proinflammatory biomarkers that are associated with pain transmission and modulation in osteoarthritis (OA) [50]. In the present study, elevated levels of CGRP were observed in the untreated OA group, which is consistent with previous reports of increased CGRP levels after OA induction in MIA rats [51]. Similarly, serum NGF levels were also found to be higher in the OA group compared to the control group. Treatment with rBM-MSCs resulted in a decrease in both CGRP and NGF levels, although these levels gradually increased over time, indicating that a single administration of MSCs may not be sufficient for therapeutic efficacy. Interestingly, pain recurrence and movement limitation were reported in OA patients who were treated with autologous MSCs six months after the first injection [52]. Many studies have explored the potential of rBM-MSCs in various applications, such as tissue engineering and osteoarthritis treatment [53,54]. While these studies have not provided a direct comparison of rBM-MSCs' effects on cartilage versus synovial tissue based on serum biomarkers, a recent study has reported anti-arthritic effects of rBM-MSCs in combination with oligosaccharides and human placental extract (HPE) on a rheumatoid arthritis (RA) model in rats. The results of this study indicate significant reductions in serum levels of inflammatory cytokines and improvements in histopathological analysis of bone sections. These findings suggest beneficial effects on articular structures in general, which include both cartilage and synovial tissues [55].

IPA and MAP tools were utilized to further support the findings of our study, identifying the genes involved in the development of connective tissue, proliferation of chondroblasts, maturation, and differentiation, as well as the probable signaling mechanisms, which are consistent with previous studies [30,32]. The use of IPA and MAP tools can be instrumental in understanding the molecular mechanisms by which rBM-MSCs may be used to treat OA. IPA is a bioinformatics tool that allows researchers to analyze data from gene studies to identify biological pathways, while MAP tools predict the activity of molecules based on their structure and known biological activities [2]. Together, these tools could provide valuable insights into the molecular pathways and activities that underpin the therapeutic potential of BM-MSCs in OA, although further research would be required to realize this potential fully.

## 5 Conclusions

OA is a persistent and debilitating condition characterized by joint inflammation and pain that is currently not fully managed by current therapeutics. As a result, the use of stem cells from multiple sources, including rBM-MSCs, is being explored. In this study, we found that administering cultured rBM-MSCs intra-articularly into OA knees of rats was an effective method for partial cartilage repair. Significantly, levels of biomarkers associated with inflammation and/or cartilage degradation, which are elevated in OA, were reduced following treatment with rBM-MSCs. Despite the observation that rBM-MSCs treatment modified the disease profile and contributed to the cartilage repair, the mechanism behind this is not fully understood and could be atributed to the paracrine effects of the rBM-MSCs. An addition of another group of nmormal rats with rBM-MSCs would help cross-verify the observed differences biochemical parameter and would have strengthed our findings. Moreover, only a long-term study can ascertain the nature of the newly formed cartilage, whether it remains as articular cartilage becomes transformed as a fibrocartilage. In general, the limitations that surrond the use of BM-MSCs for the treatment of OA include the need for further clinical validation, the complexity of the disease which may require a multifaceted treatment approach, and the critical balance of cellular interactions that must be maintained for effective treatment outcomes. As such, MSCs demonstrate promise as a therapy for patients, particularly those with advanced OA. Furthermore, evaluating OA-related serum biomarkers may aid in assessing the therapeutic efficacy of MSCs.

Supplementary Materials: Table S1: the genes identified using Ingenuity Pathway Analysis (IPA) that are associated with mesenchymal stem cells and play a key role in osteoarthritis (OA).; Table S2: list of genes involved in biological functions related to chondrogenesis, connective tissue cell development and differentiation, and osteoblast growth and differentiation.

## CRediT authorship contribution statement

**Abdulwahab Noorwali:** Writing – review & editing, Writing – original draft, Supervision, Resources, Project administration, Investigation, Formal analysis, Conceptualization. **Fadwa Aljoud:** Visualization, Validation, Software, Methodology, Formal analysis, Data curation. **Amani Alghamdi:** Validation, Methodology, Investigation, Formal analysis, Data curation. **Noora Sattami:** Visualization, Validation, Resources, Methodology, Formal analysis, Data curation. **Taghreed Bashah:** Visualization, Validation, Resources, Methodology, Formal analysis, Data curation. **Abdulsalam Noorwali:** Visualization, Validation, Resources, Methodology, Investigation, Formal analysis, Data curation. **Peter Natesan Pushparaj:** Writing – review & editing, Visualization, Software, Resources, Conceptualization. **Kalamegam Gauthaman:** Writing – review & editing, Writing – original draft, Resources, Investigation, Formal analysis, Data curation, Conceptualization.

## Institutional review board statement

This study was approved by the Bioethics Committee of King Abdulaziz University (KAU), Jeddah, Saudi Arabia, under protocol number (374-16). All animal experiments were performed in accordance with the guidelines of our institution and Animal Welfare Committee.

## Data availability statement

Data will be made available on request.

## Funding

This research received no external funding.

## Declaration of competing interest

The authors declare that they have no known competing financial interests or personal relationships that could have appeared to influence the work reported in this paper.
